# A clinical genetic method to identify mechanisms by which pain causes depression and anxiety

**DOI:** 10.1186/1744-8069-2-14

**Published:** 2006-04-19

**Authors:** Mitchell B Max, Tianxia Wu, Steven J Atlas, Robert R Edwards, Jennifer A Haythornthwaite, Antonella F Bollettino, Heather S Hipp, Colin D McKnight, Inge A Osman, Erin N Crawford, Maryland Pao, Jemiel Nejim, Albert Kingman, Daniel C Aisen, Michele A Scully, Robert B Keller, David Goldman, Inna Belfer

**Affiliations:** 1 Clinical Pain Research Section, Division of Intramural Research, National Institute of Dental and Craniofacial Research, National Institutes of Health, DHHS, Bethesda, MD, USA; 2 Statistics Core, Division of Population and Health Promotion Sciences, National Institute of Dental and Craniofacial Research National Institutes of Health, DHHS, Bethesda, MD, USA; 3 General Medicine Division and the Clinical Epidemiology Unit, Medical Services, Massachusetts General Hospital, Harvard Medical School, Boston, MA, USA; 4 Department of Psychiatry and Behavioral Sciences, Johns Hopkins University School of Medicine, Baltimore, MD, USA; 5 Office of the Clinical Director, National Institute of Mental Health, National Institutes of Health, DHHS, Bethesda, MD, USA; 6 Laboratory of Neurogenetics, National Institute on Alcohol Abuse and Alcoholism, National Institutes of Health, DHHS, Rockville, MD, USA; 7 Howard Hughes Medical Institute, Bethesda, MD, USA; 8 Maine Spine and Rehabilitation, Portland, ME, USA

## Abstract

**Background:**

Pain patients are often depressed and anxious, and benefit less from psychotropic drugs than pain-free patients. We hypothesize that this partial resistance is due to the unique neurochemical contribution to mood by afferent pain projections through the spino-parabrachial-hypothalamic-amygdalar systems and their projections to other mood-mediating systems. New psychotropic drugs for pain patients might target molecules in such brain systems. We propose a method to prioritize molecular targets by studying polymorphic genes in cohorts of patients undergoing surgical procedures associated with a variable pain relief response. We seek molecules that show a significant statistical interaction between (1) the amount of surgical pain relief, and (2) the alleles of the gene, on depression and anxiety during the first postoperative year.

**Results:**

We collected DNA from 280 patients with sciatica due to a lumbar disc herniation, 162 treated surgically and 118 non-surgically, who had been followed for 10 years in the Maine Lumbar Spine Study, a large, prospective, observational study. In patients whose pain was reduced >25% by surgery, symptoms of depression and anxiety, assessed with the SF-36 Mental Health Scale, improved briskly at the first postoperative measurement. In patients with little or no surgical pain reduction, mood scores stayed about the same on average. There was large inter-individual variability at each level of residual pain. Polymorphisms in three pre-specified pain-mood candidate genes, catechol-O-methyl transferase (COMT), serotonin transporter, and brain-derived neurotrophic factor (BDNF) were not associated with late postoperative mood or with a pain-gene interaction on mood. Although the sample size did not provide enough power to persuasively search through a larger number of genes, an exploratory survey of 25 other genes provides illustrations of pain-gene interactions on postoperative mood – the mu opioid receptor for short-term effects of acute sciatica on mood, and the galanin-2 receptor for effects of unrelieved post-discectomy pain on mood one year after surgery.

**Conclusion:**

Genomic analysis of longitudinal studies of pain, depression, and anxiety in patients undergoing pain-relieving surgery may help to identify molecules through which pain alters mood. Detection of alleles with modest-sized effects will require larger cohorts.

## Background

Decades of cross-sectional surveys have shown that chronic pain, depression, and anxiety often coexist. However, data derived from a single time point is consistent with diverse causal links [1] e.g., that (1) pain causes mood or anxiety disorders; (2) these affective disorders increase pain; (3) a common biological predisposition underlies both pain and affective disorders; or (4) pain or affective disorder do not directly cause the other but frequently associate with a "true" causal variable such as somatization, occupational or social stress, or ineffective coping style.

Recent reports have more directly examined the direction of causation by assessing pain and mood over time in thousands of individuals. In primary care practices and diverse occupational settings, mood or anxiety disorder at baseline predicts the subsequent onset of any chronic pain syndrome [2]; chronic widespread pain [3]; or chronic low back [4], neck [5], abdominal [6] or shoulder, arm, or knee pain [7]. Chronic pain at baseline predicts later anxiety or depressive symptoms [2]. Treatment of depression in patients with osteoarthritis reduces pain one year later [8].

Although these studies have strengthened the evidence for bidirectional causal links between pain and mood, the designs are not suited for inferring physiological mechanisms. A crucial limitation is that idiopathic "central pain amplification" or "multisomatoform" conditions [9] were mixed with conditions in which a measurable structural injury dominates the pain phenotype. To optimize mechanistically-oriented clinical studies one should collect relatively homogeneous patient samples and assess putative physiological mediators. Predominantly structurally determined pains may have a different causal relationship to mood than multisomatoform pains. Cohorts with pain caused by common and measurable structural lesions like acute surgical wounds or degenerative joint disease would be expected to resemble the general population in prevalence of previous affective disorder. In patients with multisomatoform pains, however, lifetime prevalence of depressive and anxiety disorders is triple that of the general population [10]. In these patients, some feature of brain physiology may predispose to both pain and mood disorders, and it may be more challenging to tease out causal relations between pain and affect.

**Figure 1:**
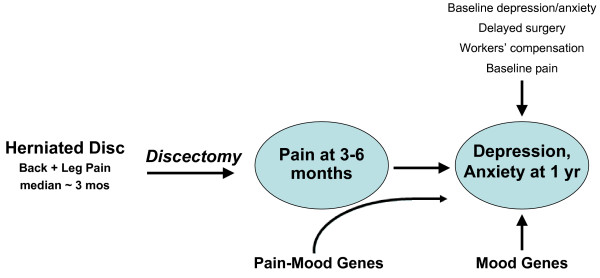
**Hypothesis regarding variability in depression and anxiety observed one year after lumbar discectomy**. Two types of genetic contribution to one-year mood scores are shown. (1) ***Mood genes ***currently studied by biological psychiatrists may contribute to the late mood effects of a stressful surgical illness. Because the diagnosis and treatment are shared by all participants, and one can measure residual pain and surgical delay as "environmental variables" and correct for their effect on moo, this design may enhance the sensitivity to detect gene effects, compared to designs that study affective disorders in patients with widely varying life stressors. These influences would show up in the statistical analysis as main effects on late mood. (2) ***Pain-mood genes ***may alter the direct effects of pain upon mood, possibly by effects on signaling molecules in the dense connections between spinal pain afferent inputs and mood-processing brain structures such as hypothalamus, amygdala, nucleus accumbens, medial orbital cortex, and cingulum. These gene effects would vary with the amount of residual chronic pain after surgery; i.e., they would show up as significant interactions between gene and pain levels in their effects upon mood.

We propose a method to investigate the following hypothesis: *Depression and anxiety triggered or worsened by pain are mediated by anatomical and neurochemical links that differ in part from those mediating depression and anxiety disorders that occur independent of pain*. A corollary is that optimal treatment of the pain patient's mood disorder might require different types of antidepressant or anxiolytic drugs than those effective in pain-free patients. This hypothesis is based on the neuroanatomical finding that spinal cord and brainstem pain-signaling neurons project via the parabrachial and solitary nuclei to densely innervate the hypothalamus, amygdala, nucleus accumbens, medial orbital cortex, cingulum, and other brain structures mediating mood [11], and the clinical observation that the presence of pain renders depressed patients relatively resistant to antidepressant drugs [12]. We predict that genetic analysis of inter-individual variability of pain-related mood change (Fig. 1) will identify novel therapeutic targets in these neural connections, whose molecular components are just beginning to be defined [13].

An economical approach to searching for these molecular mediators is to piggyback on existing longitudinal studies of painful diseases caused by definite structural lesions that include serial pain measurements and standard quality of life questionnaires. Most such questionnaires assess depression and anxiety. Perturbations of affect on these measures, although not diagnostic of clinical disorder, provide a convenient assessment of these negative emotions. To pursue this approach, we collected DNA from former participants in a large study of surgical and non-operative treatment of sciatica caused by intervertebral disc herniation [14,15]. Discectomy variably relieves patient's pain with effects occurring soon after a uniform time point, creating a quasi-experimental design for testing the effects of pain on later mood. In this sciatica study and others ([16] and RR Edwards et al., in preparation), baseline mood accounts for only a small component of the pain relief afforded by discectomy, so one can get a clearer look at the effect of pain on later mood.

In this paper we present a descriptive analysis of mood during the year following surgery, and illustrate a method for detecting genetic polymorphisms that predispose to pain-influenced mood and or anxiety disorders. This approach adapts a gene-environment interaction model that has been proposed to facilitate the detection of genes that predispose to psychiatric disorders in the presence of specific quantified stressors, such as the interaction of serotonin transporter polymorphisms and life stress to influence depression [17], and of monoamine oxidase polymorphism and parental abuse to influence conduct disorder [18]. In the current paper, we are defining the lumbar spine, nerve root, and its pain input to the central nervous system as part of the "environment" in which the brain generates an affective state.

## Methods

### Patients

Participants were members of the sciatica group of the Maine Lumbar Spine Study (MLSS [14]), a prospective cohort study conducted by approximately half of Maine's orthopedists and neurosurgeons who actively treat spine disease [19]. Patients were enrolled between 1990 and 1992, and surgical or nonsurgical treatment was determined by clinician judgment and patient preference. Patients completed questionnaires at study entry, after 3, 6, and 12 months, and then annually through year 10. Individuals who initially embarked on nonsurgical treatment but elected surgery at or before month 6 were included in the surgical group because recent data was available to serve as a baseline. Patients who crossed over to surgery after month 6 were not included in this study because the "baseline" may have occurred 6–12 months preoperatively. After completion of the 10-year study, the NIDCR and MLSS investigators developed a collaboration to collect DNA from consenting patients, under a protocol approved by the NIDCR Institutional Review Board. Of the 277 patients treated surgically, 162 contributed DNA. We also collected DNA from 118 patients treated nonsurgically and included them in our analysis of pain and mood scores at baseline. We did not include nonsurgical patients in genetic analyses of late mood change because this group was smaller, often lacked confirmatory spine imaging studies, and was probably more heterogeneous with regard to underlying pathology and treatment. Moreover, their changes in pain during the early months of the study were much smaller and more gradual than the surgical group [15]. This temporal course does not offer as clear an experimental model as the abrupt one-time surgical perturbation of pain.

### Pain measure

The primary measure of pain for this pain-mood study was the Bodily Pain intensity item on the Short-Form-36 (SF-36) quality of life instrument [20] at baseline, 3, and 6 months. Patients responded to the question "How much bodily pain have you had during the past 4 weeks?" by choosing from "very severe," "severe," "moderate," "mild," "very mild," and "none." This has been a standard scale used in analgesic clinical trials for more than 50 years. As part of the SF-36, the scale has been shown to be reliable and to be sensitive to changes in pain produced by joint replacement [20].

### Depression and anxiety measures

The mood measure was the Mental Health (MH) subscale of the SF-36 health survey. This subscale includes three Likert scale items about the frequency in the previous month of depressed vs. happy moods, and two items about the frequency of anxious vs. peaceful moods, each with 6 possible responses ranging from "all of the time" to "none of the time." Because depressive and anxious symptoms usually coexist in medically ill patients, the developers of the scale combined the items into a single score, which correlates closely with DSM-IV psychiatric diagnoses [21].

### Choice of mood candidate genes

In order to control the false-positive error rate in this modestly sized sample, prior to data analyses we chose three high-priority candidate polymorphisms that we predicted would be associated with pain-related mood deterioration:

(1) The *met *allele at the val158met polymorphism in the catechol-O-methyltransferase gene (*COMT*) reduces the ability of the enzyme to metabolize catecholamines, and has been associated with variability in an experimentally evoked pain threshold and unpleasant pain-related affect [22] and with anxiety disorders [23].

(2) The short allele in the intron 2 tandem repeat polymorphism of the serotonin transporter gene (*SLC6A4*) lowers the level of expression of the transporter protein [24] and alters cerebral processing of fear stimuli [25]. This allele has been associated with neuroticism and the risk of lifetime major depression [26]; and with depression related to stressful life events [18].

(3) The met allele of the val66met polymorphism in the brain-derived neurotrophic factor gene (BDNF) lowers activity-induced secretion of this trophic factor. The met allele has been associated with geriatric depression [27] and anxious temperament [28].

In addition to the *a priori *selection of three putative mood genes, we carried out exploratory analyses of polymorphisms in 25 additional genes that we had previously genotyped for pain genetics studies. We recognized that correction for multiple testing a cohort of several hundred patients [29] might render these analyses suitable only for generating hypotheses for future study. The genes were: galanin; galanin receptors 1, 2, and 3; interleukin (IL)-1α and β; IL-1 receptor antagonist; IL-6; IL-10; IL-13; tumor necrosis factor α; adrenergic receptors 2A, 2B, and 2C; mu opioid receptor; glial cell derived neurotrophic factor (GDNF); tyrosine hydroxylase; kainate-3 glutamate receptor; downstream regulatory element antagonistic modulator (DREAM); bradykinin receptors B1 and B2; chemokine receptor 5; purinergic receptor ligand-gated ion channel P2X4; calcium channel, voltage-dependent, α2/δ subunit 2; and chemokine (C-X-C motif) ligand 5 (ENA-CXCL5).

### Genotyping methods

#### SNP markers

The physical position and frequency of minor alleles (>0.05) from a commercial database (Celera Discovery System, CDS) were used to select SNPs spaced at 2–5 kb intervals throughout each gene region plus 4–6 kb upstream and 4–6 kb downstream of each gene. Allele frequencies of markers and their locations in the genes described in the Results appear in Additional file 1.

#### Genomic DNA

Genomic DNA was extracted from lymphoblastoid cell lines and diluted to a concentration of 5 ng/μl. Two-μl aliquots were dried in 384-well plates.

#### Polymerase Chain Reaction (PCR) amplification

Genotyping was performed by the 5' nuclease method [30] using fluorogenic allele-specific probes. Oligonucleotide primer and probe sets were designed based on gene sequences from the CDS. Reactions were performed in a 5 μl volume containing 2.25 μl TE (ABI Assays On Demand) or 2.375 μl TE (ABI Assays By Design), 2.5 μl PCR Master Mix (ABI, Foster City, CA), 10 ng genomic DNA, 900 nM of each forward and reverse primer, and 100 nM of each reporter and quencher probe. DNA was incubated at 50°C for 2 min and at 95°C for 10 min, and amplified on an ABI 9700 device for 40 cycles at 92°C (ABI Assays on Demand) or 95°C (ABI Assays By Design) for 15 s and 60°C for1 min. Allele-specific signals were distinguished by measuring endpoint 6-FAM or VIC fluorescence intensities at 508 nm and 560 nm, respectively, and genotypes were generated using Sequence Detection System V.1.7 (ABI). Genotyping error rate was directly determined by re-genotyping 25% of the samples, randomly chosen, for each locus. The overall error rate was <0.005. Genotype completion rate was 0.96.

#### Inference of haplotypes

Haplotype phases – i.e., how the directly measured SNP alleles were distributed into two chromosomes in each patient – were inferred by the expectation-maximization (EM) algorithm (SAS/Genetics, Cary, North Carolina, USA).

#### Statistical analysis

We specified as our primary analysis the test of the interaction between a (1) particular genetic polymorphism and (2) the mean bodily pain at the 3 and 6 month postoperative time points in predicting the SF-36 MH at 12 months. The secondary analysis was the same analysis carried out on the baseline SF-36 MH score, examining the effect of genotype, baseline pain score, and their interaction. This latter analysis included patients who elected non-surgical treatment as well. If there is no interaction, the functions describing bodily pain as a contributor to mood (i.e., regression of mood on bodily pain) are the same across the different SNP genotypes. The initial selection of covariates from the MLSS dataset was based upon the chronic pain literature and winnowed by modeling of the data prior to analyzing for genotypes. We pre-specified the additive model as most plausible for the effects of 0, 1, or 2 copies of a polymorphism on mood but also carried out analyses of dominant and recessive models.

## Results

### Relation between pain and mood

#### Baseline pain and mood

**Figure 2:**
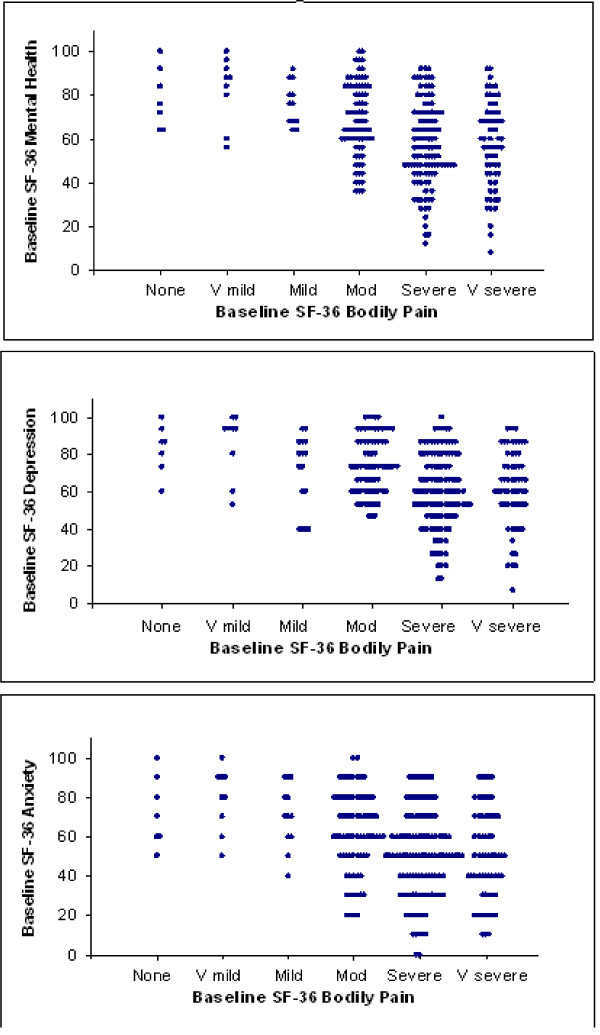
**Baseline mood vs. pain in 277 patients with subacute sciatica, regardless of subsequent surgical or nonsurgical treatment**. Overall intensity of "bodily pain" over the month before seeing a surgeon for sciatica explains about 13% of the variance in depressive and anxious feelings over the same period (p < 0.0001), assessed by the five-item SF-36 Mental Health Subscale (*top panel*). Higher scores on the y axis correspond to better mood. The other panels show similar relations to pain of the three items of the subscale pertaining to depressed mood (*middle*) and the two items pertaining to anxiety (*bottom*).

Fig 2 shows the relation at study baseline between the SF-36 bodily pain intensity question and the SF-36 MH subscale (*top panel*) and its items assessing depression (*middle*) and anxiety (*bottom*) in 276 patients, including those who subsequently underwent surgical or non-surgical treatment. Consistent with the literature, patients' scores on anxiety and depression items were closely correlated (r = 0.68, p < 0.001). Table 1 shows the results of a regression analysis of contributions to the variance in baseline symptoms of depression and anxiety. High baseline pain (about 13% of variance), receiving workers' compensation (5% of variance), prolonged pain prior to presentation (3% of variance), and younger age (1% of variance) were significantly associated with more symptoms of depression and anxiety over the month before presentation.

**Table 1:** Contributions of the variables to baseline SF-36 Mental Health scores

			Correlation (r) with			
*Quantitative variables*	Mean	STD	Baseline SF36-MH		R^2^	p-value

Baseline bodily pain	3.67	1.12	-0.36		0.130	<0.0001
Age	41.8	10.6	0.15		0.023	0.0121

			Baseline SF36-MH			
*Categorical variables*	Category	n	Mean	STD		

Sex	Male	166	61.25	19.88	0.002	0.5065
	Female	110	62.95	19.35		
Workers' compensation	Yes	105	56.15	20.53	0.052	0.0001
	No	171	65.47	18.27		
Length of episode	<=6 week	54	68.52	18.99	0.033	0.0109
	6–26 weeks	133	61.29	17.95		
	>26 weeks	89	58.88	21.68	0.006	0.2024
Comorbid illnesses	Yes	69	59.36	19.81		
	No	207	62.78	19.58		

	R^2 ^from the model including all above variables				**0.227**	

Note: R^2 ^was calculated using the model including only one variable.

SF-36 MH scores range from 0–100, where 100 means "always happy and calm over past month."

280 (surgical+ non-surgical) patients have DNA, four patients, 3 missing baseline SF36-MH and 1 missing baseline bodily pain, were dropped from analysis

In covariate selection, linear regression with backward selection method (p = 0.1) was applied, where baseline Sf36-MH was dependent variables, age, sex, workers' compensation, baseline bodily pain, comorbid illnesses, patient group (nonsurgical vs. surgical), marital status, education, and prior episodes were independent variables, and age, sex and workers' compensation were fixed in the model

#### Time course of mood after successful or unsuccessful discectomy

**Figure 3:**
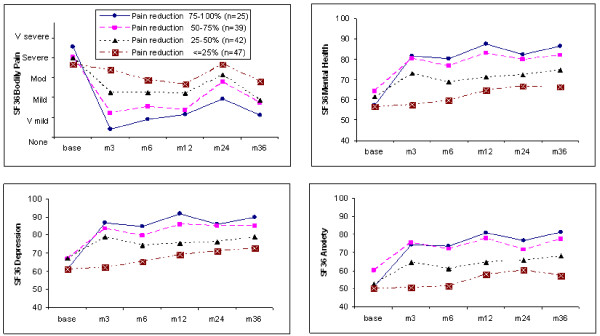
**Pain and mood over time after surgical discectomy**. In each panel, the four curves represent subgroups of 153 surgical patients divided according to the percentage reduction in "bodily pain" from the baseline to the three month rating. *Top left: *Bodily pain plotted against time over three years postoperatively. *Top right: *SF-36 Mental Health scores plotted against time. Higher values on the y axis correspond to less depressed or anxious feelings. Note that mood sharply improves at the first postoperative point in the three subgroups of patients with 25–100% reduction in pain, but mood does not worsen in the group with minimal pain relief. *Bottom: *The items specifically related to depression (*left*) and anxiety (right) show similar relations to pain reduction.

153 surgical patients provided baseline and 3 month questionnaires and DNA. We divided these patients into four groups according to the percent reduction they reported on the 0–5 point SF-36 bodily pain intensity from pre-surgical baseline to the first postoperative observation at 3 months: 75–100%, 50–75%, 25–50%, and ≤ 25%. Fig 3a (*top left*) shows the time course of bodily pain intensity over 3 years for the four quartiles. Fig 3b (*top right*) shows the time course of SF-36 MH; and Figs 3c and 3d (*bottom*) break this down into the items of the SF-36 MH relating to depression and anxiety, considered separately.

The baseline points in Figs 3b–d show little difference in mood between the groups at baseline, suggesting that factors other than baseline mood account for most of the large inter-individual differences in surgical relief of pain. At the three-month time point, the three groups of patients with the greatest reduction of pain from baseline reported an immediate improvement in mood. Surprisingly, the quartile of patients with the least improvement in pain reported, on average, a small improvement in mood from 6 to 24 months.

**Figure 4:**
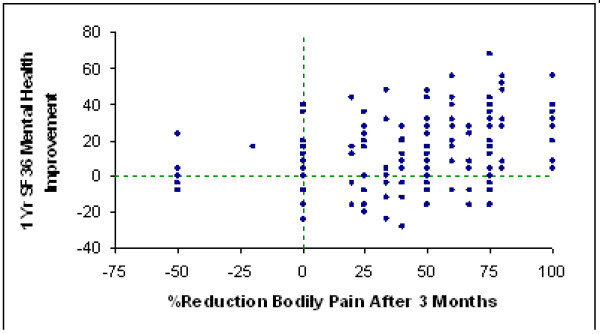
**Individual variation in improvement in mood over first year after discectomy plotted against the percent of bodily pain reduction produced by surgery**. Each point represents one patient.

However, patients demonstrated considerable variability in the relationship between pain and mood. Fig 4 shows that there is a large amount of individual variability in mood change from baseline to one year at each level of acute surgical pain improvement. Table 2 shows the results of a regression analysis of SF-36 MH scores at 12 months. More intense pain at the 3 and 6 month time points (about 20% of variance), baseline depression and anxiety (19% of variance), "crossing over" to surgery several months after choosing nonsurgical treatment at baseline (8% of variance), receiving workers' compensation at baseline (4% of variance), and more intense pain at baseline (1% of variance) were associated with more anxiety and depression at 12 months.

**Table 2:** Contributions of the variables to 1-year SF-36 Mental Health scores

			Correlation (r) with			
*Quantitative variables*	Mean	STD	1 year SF36-MH		R^2^	p-value
	
Mean bodily pain at 3 and 6 month	2.03	1.11	-0.450		0.203	<0.0001
Baseline SF36-MH	61.62	18.15	0.436		0.190	<0.0001
Baseline bodily pain	3.9	0.99	0.118		0.014	0.1642
Age	42.84	10.07	0.042		0.002	0.623
	
			1 year SF36-MH			
*Categorical variables*	Category	n	Mean	STD		
	
Sex	Male	86	74.88	20.34	0.001	0.724
	Female	55	73.60	21.92		
Workers' compensation	Yes	42	68.00	25.05	0.040	0.018
	No	99	77.09	18.44		
Crossover	0 month	120	76.83	18.54	0.079	0.001
	3 or 6 month	21	60.38	28.03		
	R^2 ^from the model including all above variables				**0.435**	

Note: R^2 ^was calculated using the model including only one variable.

In covariate selection, linear regression with backward selection method (p = 0.1) was applied, where 1 year SF-36 MH was the dependent variable; age, sex, workers' compensation, baseline bodily pain, prior episodes, and the SF-36 General Health, Vitality, Social Function, and Emotional Role subscales were independent variables; and age, sex and workers' compensation were fixed in the model.

141 of 162 surgical patients with both 1 year mood data and all 7 covariates and DNA were used in the 1 year mood analysis.

"Crossover" refers to the time point when a questionnaire showed that a patient who initially chose nonsurgical treatment underwent lumbar discectomy.

### Genetic analysis of relation between pain and mood

**Figure 5:**
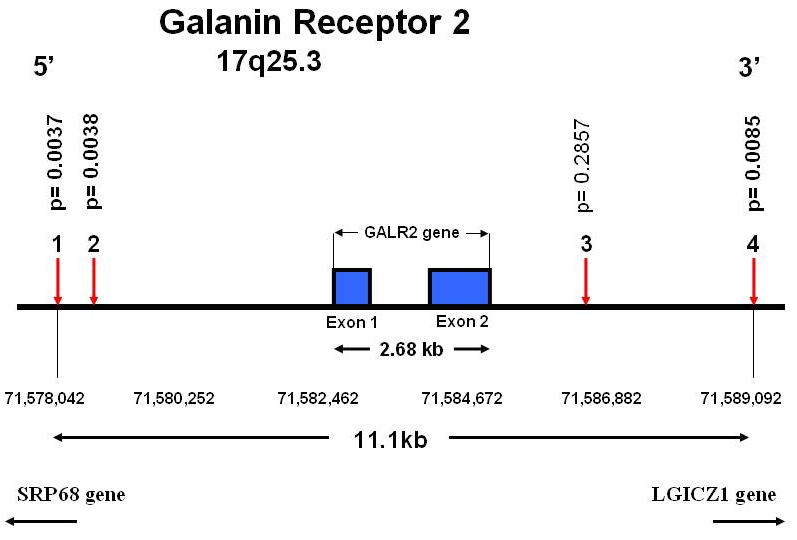
**Galanin-2 receptor gene**. Physical locations of the four genotyped single nucleotide polymorphisms (SNPs). Coding exons are shown as solid blocks. SNP locations are from the SNP Browser software and the Panther Classification System public database, February, 2006. P values for the effect on one-year SF-36 Mental Health scores of the *SNP x 3–6 month pain *interaction term are shown above each SNP.

**Figure 6:**
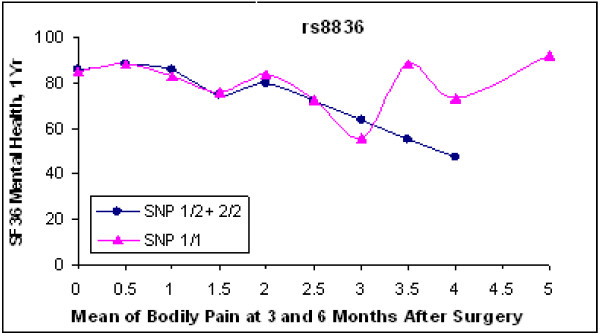
**Pattern of interaction between galanin-2 receptor SNP and residual pain upon 12-month mood score**. SNP rs8836 showed a significant interaction term in the recessive model (p = 0.003, uncorrected for multiple tests). The 12 month SF-36 MH mood scores are plotted against the mean of bodily pain scores at 3 and 6 mos. Each point is the mean 12-month mood for all of the patients with that bodily pain score and genotype. The curve connecting the triangles represents 33 patients homozygous for the uncommon allele; the curve connecting the filled circles represents 93 patients with one or no copies of the uncommon allele. In the presence of high residual postoperative pain, 2 copies of the uncommon allele appear to be associated with relative protection against symptoms of depression and anxiety, but the small numbers of such patients make this result illustrative, not statistically persuasive.

Chi-square tests showed that all SNPs used in the study were in Hardy-Weinberg equilibrium. No polymorphism in any of the three prespecified mood candidates, COMT, BDNF, and 5HTT had a significant main effect on 12-month mood, or a significant gene-pain interaction on mood. None of the other 25 genes we examined showed a strong enough association with the mood endpoint to remain significant after correction for the multiple candidate genes, and where appropriate, for multiple analysis models, or multiple SNPs within one gene. In order to illustrate the method, however, we show the results for the galanin-2 receptor. Three of the four SNPs tested in the gene (Fig. 5) showed uncorrected *p *values of 0.003 to 0.008 for a recessive model of interaction with 3–6 month pain to explain variance in 12-month mood. An analysis of a haplotype incorporating these SNPs showed a nominally significant interaction term (p = .01). However, a nominal p value < 0.001 would have been necessary to correct for the 28 candidate genes and multiple analysis models. With that caveat, we suggest that Fig. 6 illustrates a pattern consistent with a neurochemical interaction between pain and mood processing. In patients with 3–6 month pain levels of none to moderate (0–3), GAL2R genotype did not appear to affect 12-month mood. In patients with residual pain that was moderate/severe, severe, or very severe (3.5–5), patients with one or two copies of the common allele of this SNP had greater emotional distress (i.e., lower SF-36 Mental Health scores) than patients homozygous for the uncommon allele.

#### Baseline mood scores

**Figure 7:**
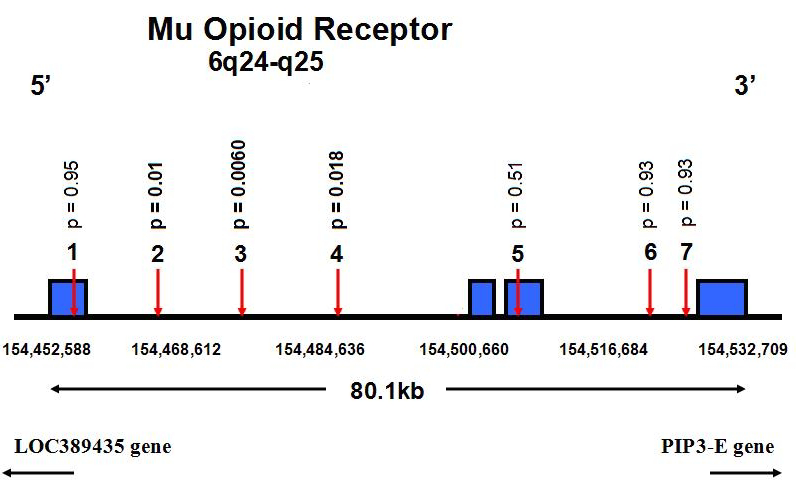
**Mu opioid receptor gene**. Physical locations of the eight genotyped single nucleotide polymorphisms (SNPs). Coding exons are shown as solid blocks. SNP locations are from SNP Browser software and the Panther Classification System public database, February, 2006. P values for the effect on one-year SF-36 Mental Health scores of the *SNP x 3–6 month pain *interaction term are shown above each SNP. SNP #1 m tge wekk-known Asn 40 Asp polymorphism, was not associated with mood scores.

**Figure 8:**
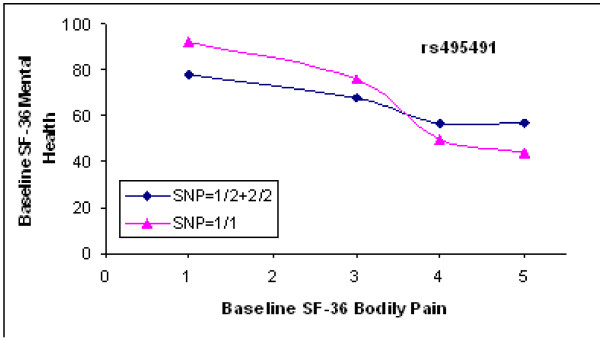
**Pattern of interaction between mu opioid receptor SNP and baseline pain upon baseline mood score**. SNP rs495491 was one of the three SNPs that showed a significant interaction term in the recessive model (p = 0.003, uncorrected for multiple tests). The baseline SF-36 MH mood scores are plotted against the baseline bodily pain scores. Each point is the mean baseline mood for all of the patients with that bodily pain score and genotype. The curve connecting the triangles represents 17 patients homozygous for the uncommon allele; the curve connecting the diamonds represents the 252 patients with one or no copies of the uncommon allele. In the presence of high baseline pain, 2 copies of the uncommon allele appear to be associated with symptoms of depression and anxiety, but the modest sample size and multiple genes tested make this result illustrative, not statistically persuasive. Data from patients with baseline pain of 0–2 (none, very mild, or mild) were pooled because few patients had such low pain scores at presentation.

Results of genetic analyses for baseline mood were similar to those for the 12-month mood data. No polymorphism in COMT, BDNF, and 5HTT had a significant main effect on baseline mood, or a significant gene-pain interaction on mood. None of the other 25 genes showed a strong enough association with the mood endpoint to remain significant after correction for multiple testing. However, three SNPs in the mu opioid receptor gene (Fig. 7) showed nominally significant pain-gene interactions on baseline mood, with p values ranging from 0.006 to 0.02. Fig. 8 shows that at baseline levels of pain from 0 – 3 (none-moderate), the relation between genotype and mood is similar. However, patients who are homozygous for the uncommon allele at rs495491 appear more susceptible to late emotional distress at high pain levels than patients with at least one copy of the common allele.

## Discussion

These data illustrate an approach to investigating causal relationships among pain, mood, and genetic polymorphisms patients who undergo a surgical procedure that produces variable degrees of pain relief. The degree of surgical relief of pain at the first two postoperative time points explains 20% of the variance in mood at 1 year (p <0.0001), but there is also additional inter-individual variation in mood (Fig. 4), some of which may result from inherited genetic variation. Because surgical relief of pain is a large abrupt change occurring at a fixed time point, this experimental design is well-suited for studying physiological events over time.

Although the galanin-2 receptor and mu opioid receptor are plausible candidates to mediate effects of pain on mood, and showed graphical patterns consistent with a pain-gene interaction, we cannot prove these specific effects because of our modest sample size. Although this was the largest prospective study of pain from a uniform lesion that we could identify at the outset of the project, a several hundred patient cohort does not provide sufficient power to correct for the tests of dozens of genes and multiple analytical models unless the relative risk conferred by the polymorphism is more than 2.5, larger than most common polymorphisms for which a link to medical diseases have been established [29]. However, major medical centers perform thousands of many types of pain-relieving operations each year, making possible more powerful searches of this type. The number of simultaneous statistical tests supported by candidate studies increases steeply with sample size. For example, just an eight-fold increase in N permits a million-fold increase in independent tests, sufficient to examine the genome in detail [29].

Another limitation of this study is the lack of clinical diagnosis of anxiety or depressive disorder. Across cohorts of patients, the SF-36 MH subscale correlates strongly with research psychiatric diagnoses and changes with successful treatment, but cannot provide individual diagnoses [20]. The SF-36 pain measure does not have optimal precision either, for estimating the actual pain level over many months. Bellamy et al. [31] found that a 0–10 point numerical scale and 100 mm VAS were more sensitive than a 5-category pain intensity scale, while Jensen and McFarland [32] reported that the average of 7 pain measurements at different times gives a better estimate of actual pain than 1–2 measurements. Quality of life researchers are currently seeking to improve diagnostic precision with computerized adaptive algorithms that choose items to hone in on each subject's response range.

It is possible that some of patients with the most severe mood disorder may have refused to return questionnaires or contribute DNA, lessening the study's power to examine this link. Another possible gap is that in the 16 years since this study was begun, pain psychologists learned that styles of coping with pain and environmental stressors are as important determinants of many pain outcomes as mood. This study did not include detailed measurements of pain catastrophizing [33], pain self-efficacy, and stressors in the work and personal environment [34], which one might consider for a new prospective study of pain and mood.

While we cannot exclude a false positive in this study, the possible mood mediating effects of the galanin-2 and mu opioid receptor polymorphisms should be studied in additional cohorts of patients with pain. The neuropeptide galanin is widely expressed in the central nervous system, including areas regulating emotionality [35]. It has been implicated in a wide range of physiological functions including pain control and cognition and in behaviors such as anxiety and depression [36]. We recently reported that haplotypes in the galanin gene were associated with anxiety-associated alcoholic phenotypes in humans [37]. The three galanin receptor subtypes, GALR1, GALR2, and GALR3 [38], are widely distributed in mood-related brain areas such as hypothalamus, central amygdaloid nucleus, and thalamus [39] and may mediate anxiety-associated behavior [40]. No common functional polymorphism has yet been identified in the human *GALR2 *gene. There is strong linkage disequilibrium (LD) between all SNP pairs we genotyped, and on the related chromosome region (HapMap; ). Although the SNPs used in our study are not located within *GALR2*, they are within 5 kb of the start and end sites of the gene (Fig 4), and are within a haplotype block encompassing *GALR2*, its regulatory elements, and neighboring genes. The nominally significant pain-gene interaction on mood of three out of four SNPs in this region and the haplotype may reflect the contribution of a functional allele in *GALR2 *or the genes located nearby.

The mu opioid receptor is also a plausible candidate to mediate between pain and mood. The endogenous opioid system and μ-opioid receptors modulate affective behaviors [41] as well as affective components of acute pain [22]. Opioid treatment of chronic pain is often accompanied by striking improvements in mood [42]. Although the human mu opioid receptor gene (*OPRM1*) has polymorphisms that affect receptor function and are associated with some behavioral phenotypes [43], no associations with depression and anxiety disorders have yet been reported [44]. The three *OPRM1 *SNPs most closely associated with pain-related mood scores in our study are in high linkage disequilibrium and in the same haploblock, which also includes the previously reported functional non-synonymous SNP Asn40Asp (rs1799971). However, the latter SNP was not associated with mood scores. Therefore we assume that the association signal, if replicated, could be attributable to another functional allele that is still unknown.

In this study, we have tested mood effects of genes that we had already genotyped based on their involvement in chronic pain processing. For future studies on pain-mood interaction, one might select additional candidate genes reportedly associated with anxiety and depression; e.g., the genes for corticotropin-releasing hormone (CRH) [45], the adenosine A(2A) receptor [46], the dopamine D4 receptor [47], and tryptophan hydroxylase [48].

It is interesting that among patients in the quartile with the least surgical pain relief, mood did not deteriorate, and actually improved from 6 months on. Within this quartile of patients (Fig. 4), for example, pain levels at 24 months after surgery were equivalent to the presurgery levels, but mood at 24 months was considerably improved from baseline. It would be interesting to examine individual differences in resilience factors, including genetic resilience factors, that are associated with improvements in mood in the face of the surgical failure to relieve pain.

The preceding analysis of gene effects mediating mood responses to unrelieved pain in surgical patients is a gene × environment (G × E) interaction study. Thus far, the most widely cited examples of G × E interactions on behavioral endpoints [17,18] involve an environmental stress. Although our most suggestive results for the genes we examined pertained to unique pain-gene interactions on mood, the same analytic method might help to identify other genes that have a general effect on mood, demonstrated by a main effect in the regression. A potential advantage of this study design is that patients are all subject to a similar stress – several months of a painful condition followed by major surgery – and then experience variable levels of residual pain, a stressor that can be well-quantified and corrected for in the analysis. Without detailed measurements of environmental stress, it may be difficult to identify genes for depression or anxiety. For example, although twin studies suggest that the heritability of unipolar major depression disorder is 40–50% [49] it has been difficult to replicate candidate gene associations, possibly because the environmental circumstances triggering depression are so varied [50]. A major surgical procedure may provide a somewhat uniform stressful situation. Within this setting, the two factor analysis shown above may remove a large amount of variance in late mood due to pain, increasing sensitivity to a main effect of a genetic polymorphism involved in general mood-regulating mechanisms not specific to patients with pain.

It is possible that alternative statistical methods may better detect "mood" or "pain-mood" genes in the types of surgical cohort studies illustrated above. For example, patients without any pain might be excluded from the pain-mood analyses, or different time-points might be chosen for the pain and mood variables. The relative merits of alternative methods can be compared more persuasively once a robust gene effect is identified to use as a gold standard.

## Conclusion

A better understanding of the specific mechanisms linking pain and mood may open up a set of new interventions to decrease the morbidity of chronic pain conditions. Basic science studies in animals [13,50] including microarray search methods are likely to yield a long list of candidates. We have demonstrated a simple method to search for genetic polymorphisms that contribute to interindividual variation in the manner in which pain produces depression or anxiety, using cohorts of patients who have undergone a surgical procedure that variably relieves pain. Cross-correlation with the results of the types of human genetic approaches outlined here may help researchers to prioritize targets and develop treatments or prophylactic interventions for pain-specific depression and anxiety.

## Competing interests

The author(s) declare that they have no competing interests.

## Authors' contributions

MBM initiated and supervised the clinical genetics study, proposed the method, and drafted the manuscript. TW designed the specific statistical method and carried out the analyses. AK advised on statistical analyses. SJA was co-PI of the original clinical study, contributed the clinical data, and advised on the analysis. RBK was co-PI of the original clinical study, contributed the clinical data, and advised on the DNA collection. RRE and JAH advised on the analyses and interpretation of psychological data. AFB, HH, CDM, IAO, ENC, JN, DCA, and MAS did the bioinformatics work and genotyping. IB trained and supervised the bioinformatics and genotyping team, interpreted this data, and wrote the molecular parts of the Methods and Discussion. MP advised on psychiatric aspects of the manuscript. DG provided laboratory facilities, trained the authors in bioinformatics and genotyping, and advised on the methods and interpretation. All authors read and approved the final manuscript.

## Supplementary Material

Additional File 1 Galanin receptor-2 gene polymorphisms and 1 year SF-36-MH Click here for file
