# Supplementary material for: A clinical genetic method to identify mechanisms by which pain causes depression and anxiety
Source: Mol Pain. 2006 Apr 19;2:14. doi: 10.1186/1744-8069-2-14 (PMC1488826; doi:10.1186/1744-8069-2-14)
Supplement: Additional File 1 — Galanin receptor-2 gene polymorphisms and 1 year SF-36-MH [file 1744-8069-2-14-S1.doc]

Supplementary Table 1. Galanin receptor-2 gene polymorphisms and 1 year SF-36-MH (only surgical patients were used)

Supplementary Table 2. Mu opioid receptor gene polymorphisms and baseline SF36-MH (both non-surgical and surgical patients were used)

“Estimate” indicates the contribution that the genotype makes to the SF-36 Mental Health score. A negative sign indicates that the effect is to worsen mood. For the additive model, the estimate refers to the effect of each copy of the uncommon allele.
